# The state of health professions students’ self-directed learning ability during online study and the factors that influence it

**DOI:** 10.1186/s12909-023-04876-z

**Published:** 2024-01-04

**Authors:** Xiaoyue Xu, Ziyi Li, Louisa Mackay, Na Li, Yaheng Zhang, Yujie Wu, Yang Zhang

**Affiliations:** 1 School of Anesthesiology, Xu Zhou Medical University, Xuzhou, Jiangsu Province China; 2 School of International Education, Xu Zhou Medical University, Xuzhou, Jiangsu Province China; 3 School of Nursing, Xu Zhou Medical University, Xuzhou, Jiangsu Province China

**Keywords:** Online study, Health professions students, Self-directed learning ability, Professional identity

## Abstract

**Background:**

Universities have widely switched from traditional face-to-face classes to online instruction as a result of the epidemic. Self-directed learning is becoming the new norm for university students’ learning styles. The ability of health professions students to learn independently during online study directly impacts the effectiveness of online medical education. The ability to learn on their own initiative may be affected by health professions students’ professional identities, defined as their positive perceptions, evaluations, emotional experiences, and identity as professionals related to medicine. This study aimed to look into the current status and the factors that influence health professions students’ self-directed learning ability (SDLA) during online study and its relationship with professional identity.

**Methods:**

This study was conducted from September to November 2022 at a medical school in East China. An online questionnaire was used to collect participants’ status of online learning, self-directed learning ability (SDLA), and professional identity.

**Results:**

One thousand two hundred ninety-eight health professions students demonstrated intermediate self-directed learning ability during online study. In terms of teacher-student interaction (F = 14.778, *P* < 0.001), student–student interaction (F = 15.713, *P* < 0.001), and learning concentration (F = 13.424, *P* < 0.001), there were significant differences in health professions students’ self-directed learning ability. Professional identity and self-directed learning ability positively correlated (r = 0.589–0.802, *P* < 0.01). Academic atmosphere and professional identity were significant predictors.

**Conclusions:**

The self-directed learning ability of health professions students while receiving instruction online is at an intermediate level and is influenced by several factors. Developing health professions students’ professional identities can enhance their ability for self-directed learning.

**Supplementary Information:**

The online version contains supplementary material available at 10.1186/s12909-023-04876-z.

## Introduction

Knowledge in the medical field is said to be evolving at the fastest rate, and to ensure their professional capacity to efficiently manage and care for patients, healthcare professionals need to be knowledgeable about the most current medical knowledge and technology. This has increased awareness that medical education should focus on preparing students for lifelong learning [1]. Self-directed learning ability (SDLA) is a vital skill for health professions students who will be lifelong learners.

Self-directed learning has been recommended as a promising methodology for lifelong learning out of intrinsic motivations with or without assistance from others [2]. Due to the outbreak of COVID-19 pandemic in 2020, Chinese universities were the first to cancel all in-person courses and switch to virtual classrooms, as other institutions globally followed suit [3]. Since then, online self-directed learning became the new normal for college students. Online classes with no physical contact with teachers left many schoolwork tasks to students themselves. Teachers’ quality supervision role in task completion was significantly reduced, and it became impossible to understand students’ mastery of knowledge in detail to adjust the teaching arrangement for different students as needed [4]. This, therefore, increased the requirement for SDLA of health professions students.

Also, in the era of rapid development of global information technology, only by enhancing sensitivity to new information and being familiar with methods of acquiring information can health professions students cope with the innovations and changes in modern healthcare. In spite of the easy access to computers and the Internet, the information-utilizing ability of students has not been greatly improved accordingly [5]. This may be related to the lack of motivation and initiative of students in acquiring information. Some students are accustomed to receiving traditional lecture-style teaching, passively memorizing the learning content, and lack the initiative to explore relevant materials and information, which affects the quality of their learning. Medical educators should adopt effective teaching strategies to enhance the effectiveness of health professions students’ SDLA.

SDLA is affected by multiple and complex factors. Previous studies mainly analyzed environmental and personal factors, such as learning motivation, learning target, learning strategy [6], age, gender, personal confidence, future career recognition, family income, and learning resources [7]. Psychological emotions such as anxiety, depression, and stress also significantly interact with SDLA [8]. However, the association between the characteristics mentioned above and SDLA during online study has received relatively little academic attention.

According to identity theory [9], the greater the recognition of one’s own profession, the more positively motivated they will be, which in turn will produce more proactive behaviors to try to make themselves outstanding. Therefore, professional identity is deemed as closely correlated with one’s learning performance.

Professional identity among health professions students refers to the overall view of the goal, social value, and emotional experience in their medical career. It determines the career choice of health professions students and plays an essential role in their career development. Developing professional identity is a dynamic process, incorporating the profession’s knowledge, skills, values, and behaviors into personal identity. Equipped with a higher professional identity, health professions students are more likely to fulfill the responsibility of a healthcare professional and provide high-quality medical treatment in the future [10, 11]. However, no relevant research explored whether there is a direct association between professional identity and the SDLA of health professions students.

Consequently, the objectives of this study were to (1) evaluate the current state of health professions students’ SDLA and its affecting elements during online study, and (2) investigate the relationship between SDLA and students’ professional identities. This study can provide a reference for medical educators to formulate relevant measures in the post-epidemic era and explore practical ways to carry out medical education in the long run.

## Methods

### Study design

The main body of Chinese medical education system is “5 + 3” model, which encompasses 5 years of undergraduate medical education (leading to a bachelor degree) and 3 years of standardized residency training (or 3 years of professional master degree) before entering into a hospital as a healthcare worker. Undergraduate education stage is the basis in developing health professions students’ self-directed learning ability and professional identity. Therefore, we selected undergraduate health professions students as study participants.

In this survey, the inclusion criteria were (1) full-time undergraduates and (2) at least two weeks of online instruction. Those who could not participate in online learning due to objective conditions were excluded.

Using the cluster sampling technique, we randomly selected 20 per cent of students from each of the eight majors: Clinical medicine, Anesthesiology, Nursing, Public health, Management, Medical Technology, Medical Information and Engineering, and Bioscience. Our research team contacted the counsellor of each major in advance and conducted the questionnaire for students at a specific time.

This study includes 10 variables of general characteristics, 1 dimension of self-directed learning ability, and 1 dimension of professional identity, totaling 12 variables. Based on the principle that the sample size should be 5–10 times the number of variables, the sample size of this study was calculated to be 66–132 (taking into account a 10% sample loss rate). The study actually surveyed 1475 students, with the sampling frame being 7375. A total of 1298 students validly responded, and the response rate was 88%.

### Measurement

The questionnaire was categorized into three sections, namely: general characteristics (including demographic characteristics and online learning situation), self-directed learning ability, and professional identity. (An additional file shows this in more detail (see Additional file 1).

### General characteristics

The research team created the general section to gather data on the demographic characteristics including gender, grade and major, and online learning situation including online network environment, variety of resources, learning concentration, teacher-student interaction, student–student interaction and academic atmosphere.

### Self-directed learning ability

Self-directed Learning Ability (SDLA) was measured using the Self-directed Learning Ability Scale [12]. The scale has thirty items and two subscales measuring self-motivation and objective behavior. On a 5-point Likert scale ranging from 1 (completely disagree) to 5 (exactly right), each item was rated. The overall and two subscale scores were determined by averaging all items within each subscale. A higher score suggested a greater SDLA level. The Scale has been frequently used in prior studies to evaluate the SDLA among health professions students. In this study, Cronbach’s alpha for the overall scale, learning motivation subscale, and learning behavior subscale were respectively 0.979, 0.944, and 0.974, indicating that each subscale had a high internal consistency.

### Professional identity

The Professional Identity Scale was used to measure professional identity among health professions students [13]. The scale contains 38 items, which are organized into six dimensions: professional cognition (7 items), professional emotion (5 items), professional commitment (5 items), professional action (8 items), professional expectation (5 items), and professional values (8 items). The scores were rated on a 5-point Likert scale ranging from 1 (completely disagree) to 5 (exactly right). With more outstanding scores, the professional identity of health professions students develops. This scale’s Cronbach’s alpha in our study was 0.978.

### Statistical analysis

SPSS Statistics 28.0 was employed to examine the data (IBM Corp, Armonk, NY, USA). Sociodemographic characters were described using frequency and percentages. Qualitative variables were assessed using a t-test on independent samples and an analysis of variance. T-test was conducted when the categorical variable was dichotomous and ANOVA was conducted when the categorical variable was polychotomous. Self-directed learning ability was given as a mean ± standard deviation. The association between self-directed learning ability and professional identity was determined using a correlation analysis. Using multiple linear regression, the influential elements of self-directed learning ability were analyzed. All the tests had a significance level of *p* = 0.05.

## Results

### General characteristics

Table 1 presents the demographic characteristics of the 1298 participants in this study. They consisted of undergraduate students from five different grades and eight different majors, with a majority being 1st-year students and in specialty of Clinical Medicine. The female respondents slightly outnumbered the male respondents. The survey focused on students who had experienced online teaching, allowing us to examine their learning situation under online teaching paradigm. To gain further insights, please refer to see Table 2 for specific details. Most respondents were able to engage in online learning under condition of good network signal without lag. ‘General’ was the most frequent choice for the surveys on variety of resources, learning concentration, question-answering (Q&A) discussion, teacher-student interaction, student–student interaction, and academic atmosphere.

**Table 1:** Demographic characteristics of the health professions students (*N* = 1298)

Variable		N	%
Gender	Male	574	44.2
	Female	724	55.8
Grade	1st year	620	47.8
	2nd year	258	19.9
	3rd year	227	17.5
	4th year	185	14.3
	5th year	8	0.6
Major	Clinical Medicine	489	37.7
	Anesthesiology	289	22.3
	Nursing	107	8.2
	Public Health	106	8.2
	Management	107	8.2
	Medical Technology	68	5.2
	Medical Information and Engineering	52	4.0
	Bioscience	80	6.2

**Table 2:** Different online learning situations that may affect students’ SDLA (*N* = 1298)

Variable		N	%
Online network environment	Smooth	1128	86.9
	General	154	11.9
	Unsmooth	16	1.2
Variety of resources	Abundant	409	31.5
	General	697	53.7
	Lacking	192	14.8
Learning concentration	Focused	322	24.8
	General	561	43.2
	Distracted	415	32.0
Q&A discussion	More use	522	40.2
	General	619	47.7
	Less use	157	12.1
Teacher-student interaction	Frequent	364	28.0
	General	635	48.9
	Seldom	299	23.0
Student–student interaction	Frequent	372	28.7
	General	641	49.4
	Seldom	285	22.0
Academic atmosphere	Good	294	22.7
	General	649	50.0
	Poor	355	27.3

Overall, these findings shed light on the learning experiences of the surveyed students, highlighting their adaptability to online teaching and providing valuable insights into various aspects of their educational journey.

### Descriptions of health professions students’ Self-Directed Learning Ability (SDLA) during online study

As previously mentioned [12], we utilized the Self-Directed Learning Ability Scale for Medical Students to assess the self-directed learning ability of the participants in our survey. The questionnaire employed a 5-point Likert scale, with the final self-directed learning ability of respondents represented by the ‘SDL score’. A higher score indicated a stronger self-directed learning ability.

Table 3 displayed the data concerning the self-directed learning ability (SDLA) of medical students during online study. The variables of gender (*P* = 0.896), grade (*P* = 0.304), and online network environment (*P* = 0.803) did not exhibit any statistically significant associations with medical students’ SDLA. However, SDLA demonstrated significant correlations with various factors, including the availability of resources, concentration during learning, Q&A discussions, teacher-student interaction, student–student interaction, and academic atmosphere (*P* < 0.001).

**Table 3:** Health professions students’ SDLA during online study

Variable		Mean (SD)	Statistical test	P	Post-hoc Analysis
Gender	Male	3.78(0.82)	t = 0.13	0.896	
	Female	3.77 (0.68)			
Grade	1st year	3.82 (0.74)	F = 1.213	0.304	
	2nd year	3.75 (0.75)			
	3rd year	3.73 (0.73)			
	4th year	3.70 (0.77)			
	5th year	3.83 (0.41)			
Network environment	Smooth	3.77 (0.73)	F = 0.22	0.803	
	General	3.80 (0.79)			
	Unsmooth	3.69 (1.13)			
Variety of resources	1.Abundant	3.89 (0.86)	F = 7.993	0.000	1 > 2(*p* = 0.005) 1 > 3(*p* = 0.003)
	2.General	3.73 (0.66)			
	3.Lacking	3.67 (0.73)			
Learning concentration	1.Focused	3.95 (0.89)	F = 13.424	0.000	1 > 2(*p* = 0.000) 1 > 3(*p* = 0.000)
	2.General	3.73 (0.69)			
	3.Distracted	3.69 (0.66)			
Q&A discussion	1.More use	3.93 (0.80)	F = 19.881	0.000	1 > 2(*p* = 0.000) 1 > 3(*p* = 0.000)
	2.General	3.60 (0.66)			
	3.Less use	3.63 (0.74)			
Teacher-student interaction	1.Frequent	3.95 (0.87)	F = 14.778	0.000	1 > 2(*p* = 0.000) 1 > 3(*p* = 0.001)
	2.General	3.69 (0.67)			
	3.Seldom	3.74 (0.69)			
Student–student interaction	1.Frequent	3.95 (0.92)	F = 15.713	0.000	1 > 2(*p* = 0.000) 1 > 3(*p* = 0.000)
	2.General	3.70 (0.66)			
	3.Seldom	3.70 (0.63)			
Academic atmosphere	1.Good	4.04 (0.90)	F = 25.941	0.000	1 > 2(*p* = 0.000) 1 > 3(*p* = 0.000)
	2.General	3.70 (0.68)			
	3.Poor	3.69 (0.65)			

### Correlation analysis between Self-Directed Learning Ability (SDLA) and Professional Identity (PI)

Table 4 lists the scores of total and each dimension of SDLA and PI. The average score for SDLA was 3.77 ± 0.74. The dimensions of self-motivation and objective behavior had average scores of 3.99 ± 0.78 and 3.69 ± 0.76, respectively. On the other hand, the average score for PI was 3.67 ± 0.64. The six dimensions of PI, ranked from highest to lowest, were: (1) professional expectation, (2) professional emotion, (3) professional values, (4) professional behavior, (5) professional commitment, and (6) professional cognition. As shown in Table 5 The correlation between the SDLA and PI dimensions (r), there was a significant correlation between SDLA and PI.

**Table 4:** The dimensions and total scores of SDLA and PI

Variable	Dimensions	Items	Mean (SD)
SDLA	2	Self-motivation	3.99 (0.78)
		Objective behavior	3.69 (0.76)
	Total score		3.77 (0.74)
PI	6	Cognition	3.30 (0.51)
		Emotion	3.79 (0.83)
		Commitment	3.63 (0.68)
		Behavior	3.74 (0.78)
		Expectation	3.80 (0.84)
		Values	3.79 (0.68)
	Total score		3.67 (0.64)

**Table 5:** The correlation between the SDLA and PI dimensions (r)

	**PI**	**Cognition**	**Emotion**	**Commitment**	**Behavior**	**Expectation**	**Values**
SDLA	0.80^a^	0.66^a^	0.76^a^	0.59^a^	0.83^a^	0.75^a^	0.69^a^
Self-motivation	0.78^a^	0.59^a^	0.75^a^	0.65^a^	0.77^a^	0.72^a^	0.72^a^
Objective behavior	0.78^a^	0.65^a^	0.73^a^	0.56^a^	0.82^a^	0.73^a^	0.65^a^

r: Correlation coefficient

^a^Significant at 0.01

These findings provide insights into the scores and dimensions of SDLA and PI among the respondents, indicating their levels of self-directed learning ability and professional identity. The correlation identified between SDLA and PI suggests a potential link between these two constructs.

### Factors influencing health professions students’ Self-Directed Learning Ability (SDLA)

We took SDLA as a dependent variable, and seven factors that may affect SDLA (professional identity, variety of resources, learning concentration, Q&A discussion, teacher-student interaction, student–student interaction, academic atmosphere) as independent variables for multiple linear regression analysis. As shown in Table 6, professional identity and academic atmosphere were significant predictors of SDLA among health professions students (F = 185.192, *p* < 0.05).

**Table 6:** Multiple linear regression analysis for health professions students’ SDLA

Model		Unstandardized coefficient		Standard coefficient	t	Sig	Collinear statistics	
		**B**	**SD**	**Beta**			**Tolerance**	**VIF**
(constant)		0.53	0.08		6.55	0.00^c^		
PI		0.92	0.02	0.79	47.10	0.00^c^	0.96	1.04
Variety of resources	Ref: Abundant							
	General	-0.01	0.03	-0.01	-0.34	0.74	0.52	1.91
	Lacking	0.06	0.05	0.03	1.15	0.25	0.52	1.94
Learning concentration	Ref: Focused							
	General	0.02	0.04	0.01	0.42	0.67	0.35	2.90
	Distracted	0.00	0.05	0.00	0.05	0.96	0.29	3.43
Q&A discussion	Ref: More use							
	General	0.02	0.03	0.01	0.47	0.64	0.55	1.81
	Less use	-0.04	0.05	-0.02	-0.87	0.38	0.61	1.65
Teacher-student interaction	Ref: Frequent							
	General	-0.00	0.04	-0.00	-0.03	0.97	0.39	2.52
	Seldom	0.02	0.05	0.01	0.34	0.73	0.36	2.81
Student–student interaction	Ref: Frequent							
	General	0.00	0.04	0.00	0.05	0.96	0.39	2.59
	Seldom	-0.04	0.05	-.002	-0.88	0.38	0.39	2.50
Academic atmosphere	Ref: Good							
	General	-0.16	0.05	-0.11	-3.46	0.00^b^	0.29	3.51
	Poor	-0.17	0.06	-0.10	-3.05	0.00_b_	0.25	4.03
a. Dependent Variable: SDLA								

Adjusted R^2^ = 0.649, F = 185.192

^a^Significant at 0.05

^b^Significant at 0.01

^c^Significant at 0.001

## Discussion

### Self-Directed Learning Ability (SDLA) among Chinese health professions students during online study

With the widespread use of online teaching paradigm during the epidemic, the SDLA of health professions students has aroused growing awareness among educators and researchers. Researches have demonstrated that SDLA was positively correlated with students’ academic performance [14]. Scores of SDLA among health professions students can reflect their learning performance. With a total average score of 3.77 ± 0.74, the SDLA of health professions students was at a medium level in this study. Jia Y [15], Jin M [16], and other researchers have reported similar results, which indicated that the overall SDLA of health professions students in general still has room for improvement.

Among health professions students, those with learning methods that suit them have higher SDLA scores than those who do not have a learning method that suits them [17]. In conventional face-to-face classes where communication between students and teachers often happens, guidance from instructors may enable health professions students to find suitable learning methods. Meanwhile, the interaction between students could facilitate the sharing and integration of learning methods. In contrast, lack of interaction is not only detrimental to the optimization of learning methods but may also reduce students’ interest in learning [18].

 Clark R [19], Keis O [20], and other researchers also reported that teacher-student interaction and student–student interaction were important factors in the effectiveness of students’ self-directed learning. These findings are in line with ours that health professions students who have less interaction with their teachers and classmates have lower SDLA scores. Meanwhile, health professions students with abundant learning resources, a good academic atmosphere, and good learning concentration during online study have better self-directed learning abilities. There were no significant differences in terms of gender, grade, and online network environment. However, some suggest that a stable network environment is a major factor for self-directed learning effectiveness online for health professions students [21]. The SDLA scores changed by gender and health professions students in higher grades had better SDLA than those in lower grades [22].

Multiple linear regression analysis in our study has shown that academic atmosphere is a significant predictor of SDLA. A good academic atmosphere helps to improve students’ self-discipline, solving the problem of distraction and lack of concentration during online study [23]. In a relaxed learning atmosphere, students are also more able to actively speak up and discuss professional issues, and become truly self-directed individuals with the ability to learn.

### Self-directed learning ability and professional identity

According to the analysis, we found that professional identity was positively correlated with SDLA and was also an essential predictor of health professions students’ SDLA during online study. The stronger the professional identity, the higher the SDLA. Health professions students with higher professional identities can take their specialties to their hearts and make positive perceptions and evaluations, thus generating interest and pride in their medical career and stimulating learning motivation [24]. Professional identity is the foundation of academic progression. Previous studies have shown that health professions students’ professional identity positively contributes to their SDLA, motivation, and academic achievement [25, 26]. In addition, professional identity can inversely predict learning burnout, so improving health professions students’ professional identity can reduce factors that are detrimental to their self-directed learning ability, such as learning burnout [27]. Helping health professions students develop a good professional identity may lead to greater development of their SDLA. The development of professional identity is considered to be something that should continue throughout the whole medical education career [11].

So far, many medical colleges’ curriculum arrangements have been segmented and tomographic [28], and have not balanced the relationship between curriculum and occupation, resulting in a lack of professional identity among health professions students. There should be coherence between basic education and clinical practice. Professional literacy education should be integrated into curricula to improve the professional identity of health professions students. Therefore, it is recommended that universities pay attention to the education of professional identity formation for health professions students. Education of professional identity formation should be carried out from the admission stage, and pre-clinical practice stage to the clinical stage, guiding students to take positive cognition of medical career, thus motivating them to study actively. In addition, teachers should incorporate career thinking into curriculums to help health professions students establish the right professional learning goals and complete their academic missions in an autonomous manner. Furthermore, the cultivation of health professions students’ professional identity should be strengthened by guiding them to take a positive perception of the value of professional study in the whole career, and thus significantly enhancing their SDLA.

## Limitation

The sociodemographic section of the questionnaire was self-arranged which may not be comprehensive to involve factors that may influence students’ SDLA. Factors such as specialty, family income, residence, and duration of classes, were not included in this study. More objective evaluation indicators during online teaching should be explored to better understand the self-directed learning ability among health professions students. In addition, although this survey was a cross-sectional study specifically during online learning, it may be better to carry out a comparative survey when online learning was switched to face-to-face classes, thus better estimating whether online environment factors can be seen as main effect factors of one’s SDLA.

## Conclusion

In this context, we found that the self-directed learning ability among health professions students during online study was at a moderate level. Academic atmosphere and professional identity could positively predict health professions students’ self-directed learning ability. The results of this survey provide data support for medical educators to formulate relevant policies and enhance teaching and learning measures to improve the self-directed learning ability among health professions students.

### Supplementary Information

**Additional file 1.** Questionnaire.

## Data Availability

The datasets generated and analyzed during the current study are not publicly available due to ethical or privacy restrictions but are available from the corresponding author on reasonable request.

## Abbreviations

SDLA: Self-directed learning ability
PI: Professional identity
