# Supplementary material for: The state of health professions students’ self-directed learning ability during online study and the factors that influence it
Source: BMC Med Educ. 2024 Jan 4;24:25. doi: 10.1186/s12909-023-04876-z (PMC10768216; doi:10.1186/s12909-023-04876-z)
Supplement: Supplementary file 1 — Additional file 1. Questionnaire. [file 12909_2023_4876_MOESM1_ESM.docx]

**Questionnaire**

Dear friends,

Thank you very much for participating in this survey. Due to the normal lockdown during the epidemic, the school adopted the online teaching mode in the early stage. We would like to learn about students' learning conditions in recent times through this questionnaire, so as to facilitate the development and improvement of medical teaching. This questionnaire is anonymous. All your information will be protected. Please answer these questions according to your real situation. There is no right or wrong answer to any question. Thank you for your support and cooperation!

Ⅰ、Demographic characteristics

1. Gender [Single choice question] *

| ○ Male |
| --- |
| ○ Female |

2. Grade [Single choice question] *

| ○1st year |
| --- |
| ○2nd year |
| ○3rd year |
| ○4th year |
| ○5th year |

3. Major [Single choice question] *

| ○Clinical Medicine |
| --- |
| ○Anesthesiology |
| ○Nursing |
| ○Public Health |
| ○Management |
| ○Medical Technology |
| ○Medical Information and Engineering |
| ○Bioscience |

Ⅱ、The following is a survey of online learning status. Please choose the corresponding options according to your real situation during the online study at your school.

1. Online network environment [Single choice question] *

| ○Smooth |
| --- |
| ○General |
| ○Unsmooth |

2. Variety of resources [Single choice question] *

| ○Abundant |
| --- |
| ○General |
| ○Lacking |

3.Learning concentration [Single choice question] *

| ○Focused |
| --- |
| ○General |
| ○Distracted |

4. Q&A discussion [Single choice question] *

| ○More use |
| --- |
| ○General |
| ○Less use |

5. Teacher-student interaction [Single choice question] *

| ○Frequent |
| --- |
| ○General |
| ○Seldom |

6. Student-student interaction [Single choice question] *

| ○Frequent |
| --- |
| ○General |
| ○Seldom |

7.Academic atmosphere [Single choice question] *

| ○Good |
| --- |
| ○General |
| ○Poor |

Ⅲ、Descriptions below are related to medical students’ self-directed learning ability and professional identity. Please choose the options according to your actual situation.

Self-directed Learning Ability Scale for Medical Students [Matrix scale questions] *

|  | Completely disagree | Disagree | General | Agree | Exactly right |
| --- | --- | --- | --- | --- | --- |
| 1、I think it is meaningful to study medicine. Although it is more difficult than other majors, I still like to study medicine | ○ | ○ | ○ | ○ | ○ |
| 2、In order to expand my knowledge, I am willing to participate in various learning activities (such as lessons of medical skills, lectures, etc.) held by the school | ○ | ○ | ○ | ○ | ○ |
| 3、I think exam results reflect personal learning to some extent and I will not be satisfied until I get an ideal score | ○ | ○ | ○ | ○ | ○ |
| 4、I think doctors are noble, and I must lay a solid theoretical foundation for my clinical skills in the future | ○ | ○ | ○ | ○ | ○ |
| 5、I often feel that time flies when I am in class or reviewing lessons by myself | ○ | ○ | ○ | ○ | ○ |
| 6、I focus on daily review. I'm not so nervous before the exam. Sometimes I choose to play to rest my mind | ○ | ○ | ○ | ○ | ○ |
| 7、I will set short-term goals as well as long-term learning goals | ○ | ○ | ○ | ○ | ○ |
| 8、I often do preview before class so as to have a general grasp of the class content | ○ | ○ | ○ | ○ | ○ |
| 9、I pay attention to the study of the general theory, because mastering the general theory is conducive to understanding and memorizing the monographs | ○ | ○ | ○ | ○ | ○ |
| 10、When reading textbooks and other reading materials, I often use red and blue pens or other pens to make lines and marks | ○ | ○ | ○ | ○ | ○ |
| 11、In class, I always like to write down the questions that I don’t understand or the questions that come to mind for further reflection after class | ○ | ○ | ○ | ○ | ○ |
| 12、I like to keep textbooks and materials together regularly to make it easier to find them | ○ | ○ | ○ | ○ | ○ |
| 13、I can choose the time of day (morning, afternoon or evening) when I am at my best mentally according to my body clock | ○ | ○ | ○ | ○ | ○ |
| 14、I can check my study at any time, find out the problems in the study plan and make adjustments | ○ | ○ | ○ | ○ | ○ |
| 15、I read carefully and strive to understand, after the exam many students will forget knowledge, but I remember | ○ | ○ | ○ | ○ | ○ |
| 16、After the exam I felt I would do better in the subjects I reviewed with my classmates | ○ | ○ | ○ | ○ | ○ |
| Please choose ‘Disagree’ | ○ | ○ | ○ | ○ | ○ |
| 17、After clinical practice, I read medical textbooks and found myself understanding the theories better | ○ | ○ | ○ | ○ | ○ |
| 18、I find that if I review what I have learned with my roommates every night before going to bed, I will remember it more firmly | ○ | ○ | ○ | ○ | ○ |
| 19、I can get the information I need from various sources (medical books, databases, medical websites, etc.) | ○ | ○ | ○ | ○ | ○ |
| 20、I have mastered various methods to search medical literature | ○ | ○ | ○ | ○ | ○ |
| 21、I am able to select suitable and practical tutorial books from relevant medical textbooks according to the learning tasks | ○ | ○ | ○ | ○ | ○ |
| 22、I am able to identify elements of relevant medical information (such as editors, publishers, keywords, etc.) | ○ | ○ | ○ | ○ | ○ |
| 23、I like to paraphrase the textbook knowledge according to my own understanding, in my own words, rather than memorizing the words in the textbook | ○ | ○ | ○ | ○ | ○ |
| 24、I like to organize the medical knowledge I have learned and make an outline or graph to enhance my understanding and memory | ○ | ○ | ○ | ○ | ○ |
| 25、I like to use clinical cases to deepen my understanding of knowledge points | ○ | ○ | ○ | ○ | ○ |
| 26、I like independent study and independent thinking, but I also like to discuss problems with my classmates | ○ | ○ | ○ | ○ | ○ |
| 27、Some problems in the homework are difficult, I always think about it first, and then ask the teacher or classmates if I can’t do it | ○ | ○ | ○ | ○ | ○ |
| 28、I often consult the teacher after class when I don’t understand the problem | ○ | ○ | ○ | ○ | ○ |
| 29、I often communicate with other students about my study | ○ | ○ | ○ | ○ | ○ |
| 30、I have my own opinions on problems while receiving help from teachers or classmates | ○ | ○ | ○ | ○ | ○ |

Professional Identity Questionnaire for Medical Students [Matrix scale questions] *

|  | Completely disagree | Disagree | General | Agree | Exactly right |
| --- | --- | --- | --- | --- | --- |
| 1、When I entered school, I knew a lot about the medical profession | ○ | ○ | ○ | ○ | ○ |
| 2、I know my responsibilities as a doctor | ○ | ○ | ○ | ○ | ○ |
| 3、The value of being a doctor is higher than that of other general professions | ○ | ○ | ○ | ○ | ○ |
| 4、The profession of doctor is one of the best in our society | ○ | ○ | ○ | ○ | ○ |
| 5、Doctors work under high pressure and with high risks | ○ | ○ | ○ | ○ | ○ |
| 6、The current medical environment is harsh | ○ | ○ | ○ | ○ | ○ |
| 7、Doctors are worth what they get | ○ | ○ | ○ | ○ | ○ |
| 8、I think doctors are happy at work | ○ | ○ | ○ | ○ | ○ |
| 9、I like being a doctor | ○ | ○ | ○ | ○ | ○ |
| 10、Being a doctor is my ideal career choice | ○ | ○ | ○ | ○ | ○ |
| 11、I am very proud to be a medical student in the association with other majors | ○ | ○ | ○ | ○ | ○ |
| 12、I get sick when people tell me doctors are bad | ○ | ○ | ○ | ○ | ○ |
| 13、Medical education has made me more determined to become a doctor | ○ | ○ | ○ | ○ | ○ |
| 14、I would be a doctor again if I could do it all over again | ○ | ○ | ○ | ○ | ○ |
| 15、If the future of doctor career is not good, I have the desire to change | ○ | ○ | ○ | ○ | ○ |
| 16、I will choose to be a doctor after graduation | ○ | ○ | ○ | ○ | ○ |
| 17、Doctor will be my career all my life | ○ | ○ | ○ | ○ | ○ |
| 18、I will follow the news reports about doctors in the society | ○ | ○ | ○ | ○ | ○ |
| 19、I am very devoted to my study | ○ | ○ | ○ | ○ | ○ |
| Please choose ‘General’ | ○ | ○ | ○ | ○ | ○ |
| 20、I take time to think about medical problems | ○ | ○ | ○ | ○ | ○ |
| 21、I am willing to participate in lectures and other activities that will help me in my medical career | ○ | ○ | ○ | ○ | ○ |
| 22、I think I have the talent to be a doctor | ○ | ○ | ○ | ○ | ○ |
| 23、I have a solid professional study | ○ | ○ | ○ | ○ | ○ |
| 24、I have a good command of operation skills | ○ | ○ | ○ | ○ | ○ |
| 25、I do well in my studies | ○ | ○ | ○ | ○ | ○ |
| 26、I think I'm qualified to be a doctor in the future | ○ | ○ | ○ | ○ | ○ |
| 27、I am confident that I will be an excellent doctor | ○ | ○ | ○ | ○ | ○ |
| 28、I hope to make achievements in my medical career | ○ | ○ | ○ | ○ | ○ |
| 29、I am optimistic about the future of the medical industry | ○ | ○ | ○ | ○ | ○ |
| 30、I am confident about the future employment | ○ | ○ | ○ | ○ | ○ |
| 31、Being a doctor is a noble profession | ○ | ○ | ○ | ○ | ○ |
| 32、It’s great that doctors can cure people | ○ | ○ | ○ | ○ | ○ |
| 33、Doctors have a relatively high social status | ○ | ○ | ○ | ○ | ○ |
| 34、Being a doctor is very respectable | ○ | ○ | ○ | ○ | ○ |
| 35、Being a doctor is just a way of making a living | ○ | ○ | ○ | ○ | ○ |
| 36、Being a doctor enables me to live the life I want | ○ | ○ | ○ | ○ | ○ |
| 37、I think being a doctor can realize my value in life | ○ | ○ | ○ | ○ | ○ |
| 38、Patients’ satisfaction is the goal of my future work | ○ | ○ | ○ | ○ | ○ |
